# Randomised controlled feasibility trial of online group acceptance and commitment therapy for functional cognitive disorder

**DOI:** 10.1192/bjo.2025.33

**Published:** 2025-05-02

**Authors:** Norman Poole, Sarah Cope, Serena Vanzan, Aimee Duffus, Tatiana Williams, Nadia Mantovani, Jared G. Smith, Barbara Barrett, Martin Scicluna, Sarah Beardmore, Mark J. Edwards, Robert Howard

**Affiliations:** South West London and St George’s Mental Health NHS Trust, London, UK; Department of Neuropsychiatry, South London and Maudsley NHS Foundation Trust, London, UK; Institute of Psychiatry, Psychology and Neuroscience, King’s College London, London, UK; Population Health Research Institute, St George’s University of London, London, UK; Clinical Research Unit, South West London and St George’s Mental Health Trust, London, UK; King’s Health Economics, King’s College London, London, UK; South London and Maudsley NHS Foundation Trust, London, UK; Division of Psychiatry, University College London, London, UK

**Keywords:** Functional neurological disorder, neuropsychiatry, psychological treatments, randomised controlled trial, functional cognitive disorder

## Abstract

**Background:**

Functional cognitive disorder is an increasingly recognised subtype of functional neurological disorder for which treatment options are currently limited. We have developed a brief online group acceptance and commitment therapy (ACT)-based intervention.

**Aims:**

To assess the feasibility of conducting a randomised controlled trial of this intervention versus treatment as usual (TAU).

**Method:**

The study was a parallel-group, single-blind randomised controlled trial, with participants recruited from cognitive neurology, neuropsychiatry and memory clinics in London. Participants were randomised into two groups: ACT + TAU or TAU alone. Feasibility was assessed on the basis of recruitment and retention rates, the acceptability of the intervention, and signal of efficacy on the primary outcome measure (Acceptance and Action Questionnaire II (AAQ-II)) score, although the study was not powered to demonstrate this statistically. Outcome measures were collected at baseline and at 2, 4 and 6 months post-intervention, including assessments of quality of life, memory, anxiety, depression and healthcare use.

**Results:**

We randomised 44 participants, with a participation rate of 51.1% (95% CI 40.8–61.5%); 36% of referred participants declined involvement, but retention was high, with 81.8% of ACT participants attending at least four sessions, and 64.3% of ACT participants reported being ‘satisfied’ or ‘very satisfied’ compared with 0% in the TAU group. Psychological flexibility as measured using the AAQ-II showed a trend towards modest improvement in the ACT group at 6 months. Other measures (quality of life, mood, memory satisfaction) also demonstrated small to modest positive trends.

**Conclusions:**

It has proven feasible to conduct a randomised controlled trial of ACT versus TAU.

Functional cognitive disorder (FCD) is increasingly recognised as a common presentation to memory clinics,^1^ accounting for at least a quarter of patients in cognitive neurology clinics.^2^ Patients with FCD report elevated levels of distress, depression and anxiety, and their persistent cognitive symptoms affect their employment status and activities of daily living.^2^ A study found that after an average of 20 months follow-up, only one of 48 patients had developed dementia,^3^ and another reported that at 10 years follow-up, none of the 90% who were contactable showed evidence of having progressed to dementia.^4^ These findings are in keeping with the low rates of misdiagnosis seen with other functional diagnoses.^5^ As such, FCD does not usually herald a progressive neurocognitive disorder.

Despite better awareness of diagnosis, in the absence of an evidence base, professionals remain uncertain how to treat patients with FCD. Most memory clinics immediately discharge the patient back to primary care, with interventions ranging from simple reassurance to community mental health team referral.^6^ Recent consensus guidance^7^ supports a role for unspecified psychological therapies, and there is emerging evidence that interventions targeting expectations, cognitive restructuring and education about the fallibility of memory are beneficial.^8^ Nevertheless, such interventions are not widely available.

## Acceptance and commitment therapy for FCD

We developed a five-session online group intervention for people with FCD that is grounded in acceptance and commitment therapy (ACT),^9^ a third-wave cognitive–behavioural therapy. ACT can reduce distress and disability in chronic pain^10^ and chronic medical conditions^11^ and has demonstrated some effectiveness as a treatment for functional motor disorders.^12^ We consider that ACT could have a specific role in the treatment of FCD, because it is aimed at improving psychological flexibility, a transdiagnostic target that is suspected to be reduced in patients with functional neurological disorder,^13^ although empirical confirmation of this is lacking. ACT emphasises engagement in meaningful activity rather than directly attempting to modify cognitive symptoms, thoughts or emotional responses, while fostering acceptance of the symptoms as experienced by the patient.^14^ There are six core processes on which ACT is built, with the overarching goal of increasing psychological flexibility.^15^ The aim is to increase acceptance of adverse states in place of avoidance; use cognitive defusion strategies to distance the individual from their thoughts and mental states; promote the observing self through mindfulness practices; encourage awareness of the present state and intentional responding to experience and sensations; identify personally meaningful and important core values; and take committed values-driven actions even in the presence of difficult thoughts, emotions or circumstances.

We hypothesise that psychological inflexibility may manifest as metacognitive doubt and hypervigilance for cognitive failures; this is because the individual identifies strongly with the narrative that their memory always fails, so they doubt their general ability to remember accurately,^16^ in keeping with an overly precise but erroneous prior. This results in excessive checking for lapses, reassurance-seeking or over-reliance on *aide memoires*, which could, paradoxically, intensify cognitive monitoring and distress. Those with FCD may avoid situations (so-called ‘cogniphobia’^17^) that trigger anxiety, such as social functions or more challenging work roles, contributing to what is called in the ACT literature ‘secondary suffering’. Participants are encouraged to appreciate that although they have no choice about the primary suffering, the cognitive symptoms, they have agency with respect to behaviours which exacerbate secondary suffering. By fostering acceptance of cognitive lapses and shifting the focus to values-based action rather than symptom control, we suggest that ACT might help to reduce distress. Following early consultation with members of our patient and public involvement (PPI) group, some of whom had participated in pilot cohorts, we opted for an online format to facilitate engagement, as many patients with FCD fall within the working age population and continue to work.^2^ Group formats can be especially helpful for functional disorders, as they can help to improve acceptance and understanding while reducing the stigma and isolation of having a misunderstood diagnosis.^18^

## Aims

We aimed to establish the feasibility of a future randomised controlled trial (RCT) of ACT for FCD, comparing it against treatment as usual (TAU). Our specific objectives were to investigate the willingness of people with FCD to be randomised in the trial, retention of participants, rates of completion of outcome measures, acceptability of an ACT intervention for FCD, fidelity of the intervention to ACT principles, healthcare use by participants, and whether a signal of efficacy could be detected.

## Method

### Study design and setting

We conducted a parallel, two-arm, randomised feasibility study of an online ACT intervention versus a TAU control for patients with FCD. A full description of the methodology has been published previously as an open access protocol.^9^ The study took place at St George’s and South West London Mental Health Trust. Funding was provided by the National Institute for Health and Care Research (NIHR; grant number NIHR202743).

The authors assert that all procedures contributing to this work comply with the ethical standards of the relevant national and institutional committees on human experimentation and with the Helsinki Declaration of 1975, as revised in 2013. All procedures involving human participants and/or patients were approved by the South East Scotland Research Ethics Committee (reference: 22/SS/0059) and Health Research Authority (IRAS 313730). The study was registered with the ISRCTN registry (ISRCTN12939037) and the protocol is available at https://doi.org/10.1136/bmjopen-2023-072366.

### Participants

Forty-four participants were recruited from diagnostic memory, cognitive neurology and neuropsychiatry clinics in selected London services. Inclusion criteria were having an established diagnosis of FCD given by the recruiting site (according to consensus criteria^1^) and confirmed by the research team from a review of patient history and investigations, being more than 18 years old, and being able to provide written informed consent. Exclusion criteria were disabling cognitive symptoms in the context of a primary psychiatric or neurocognitive disorder, medium or high risk of self-harm, having another predominant functional disorder (e.g. functional seizures) or being unable to understand English. We had initially planned to exclude those with greater than mild–moderate depressive or anxiety disorders based on nine-item Patient Health Questionnaire (PHQ-9) score ≥ 15 and/or Generalized Anxiety Disorder (GAD7) score ≥ 15 but removed this criterion in response to participant feedback and retrospectively contacted anyone excluded previously on that basis. We reflected that it was unknown what effect severity of depressive disorder might have on engagement and response, as long as the cognitive symptoms were disproportionate to the depression; therefore, we should not exclude on the basis of a simple screening measure. Participants who met the inclusion criteria were provided with written information about the study and asked to complete the consent form and baseline outcome measures.

### Randomisation and blinding

After completing baseline outcome measures (online or paper format available for those who requested this) and providing consent to be involved in the study, participants were randomised by the trial manager (S.V.) into one of two groups, ACT + TAU or TAU alone, using a block randomisation procedure with randomly permuted block sizes of 2 and 4. Owing to the nature of the intervention, participants, the trial manager (S.V.) and treating clinicians (N.P. and S.C.) were unblinded, but the research assistants (A.D. and T.W.) collecting outcome data, the statistician (J.G.S.) (for intention-to-treat analyses) and all others involved in the trial were blinded to treatment allocation.

### Procedures

The ACT intervention was delivered online via the Microsoft Teams platform in four cohorts, with group sizes ranging from three to nine. N.P. and S.C. were co-therapists for all groups. The intervention involved five 90-min structured sessions in total, delivered over an 8-week period. The initial four sessions were delivered weekly and included mindfulness exercises, psychoeducation, exercises to increase psychological flexibility and identification of value-based goals. Over the course of these four sessions, participants were supported to develop a personalised action plan for the weeks and months ahead. The final session, scheduled for 1 month after the fourth session, was used to help participants to identify and overcome barriers to realising their action plan. More details on the intervention are available in the published protocol.^9^ Participants received a schedule of ACT sessions before randomisation and were asked to consent only if they could attend all sessions. Weekly email and/or text reminders were sent to all attendees to optimise attendance.

TAU consisted of explanation of the diagnosis, provision of written information and management of the condition as per standard clinical care. All participants received TAU, whereas half were randomised to receive ACT in addition.

### Outcome measures

The measures of feasibility were: recruitment rate, adherence to the intervention and intervention acceptability. We also selected an ACT-specific outcome measure (Acceptance and Action Questionnaire II (AAQ-II) score) to assess signal of efficacy. These feasibility criteria were predefined as set out in the published protocol.^9^

Health-related quality of life and functional disability outcome measures were: the World Health Organization Disability Assessment Schedule 2.0 (WHODAS 2.0; mean standardised domain and total scores were derived on the basis of item–response theory); the five-dimension five-level EuroQol health scale (EQ-5D-5L), with scores for evaluation of health state derived from the norm-based value set developed for populations in England;^19^ and the Investigating Choice Experiments Capability Measure for Adults (ICECAP^20^). Subjective cognitive and psychological symptoms were assessed using the Multifactorial Memory Questionnaire (MMQ^21^), PHQ-9^22^ and GAD-7.^23^ Service use, subjective improvement and satisfaction were assessed using the Adult Service Use Schedule (AD-SUS^24^); the Clinical Global Impression-Improvement Scale (CGI^25^), single item, participant rated; and a satisfaction with treatment scale (a single-item, five-point Likert scale), respectively. Outcome measures were completed at baseline and again at 2, 4 and 6 months post-randomisation. All scheduled ACT sessions were arranged to ensure that the intervention had concluded before the 2-month outcome measures were completed. Participants were paid £10 at each time point for successful completion of outcome measures.

Safety was monitored by self-report of adverse events at each time point; these were recorded and monitored by the trial manager. Any adverse event requiring immediate medical attention or hospital admission was to be coded as a serious adverse event.

### Statistical analysis

No power calculation was completed, as this was a feasibility trial. Rather, a target recruitment of 48 participants was considered sufficient to provide reliable estimates of feasibility outcomes such as recruitment, adherence and retention rates to inform the design of a fully powered RCT.^26^

Baseline characteristics were reported according to treatment arm (reported as mean (s.d.) for continuous variables that were normally distributed or median (interquartile range) if non-normal and frequency (%) for categorical variables). Feasibility outcomes were summarised using descriptive statistics and compared with full-trial progression criteria. The signal of efficacy for ACT + TAU compared with TAU was analysed according to outcome variables at 2, 4 and 6 months, using multilevel mixed-effects linear regression models on an intention-to-treat basis. These random intercept (mixed) models included intervention group, time and intervention group by time interaction and used restricted maximum likelihood estimation.^21^ For each measure, Little’s test of missing completely at random indicated that data were missing at random (for all tests, *P* > 0.391). Subsequent per-protocol analyses, considering only the data available at each time point and only those participants in the ACT + TAU group completing treatment (i.e. attending ≥4 sessions), were administered by calculating between-group differences for primary and secondary outcome measures at each follow-up time point, adjusted for pre-treatment score on the measure of interest, using analysis of covariance, which relies on complete-case analysis.

There was no emphasis on hypothesis testing, which is reserved for a future main trial. Rather, effect sizes (Hedges’ *g*) at all post-randomisation time points were calculated (with associated 95% confidence intervals to explore imprecision around effect sizes^27^) in intention-to-treat analyses by dividing the difference between adjusted pre-treatment to post-treatment mean changes in ACT + TAU and TAU participants by the pre-treatment pooled standard deviation^28^ and in per-protocol analyses by dividing the difference between (adjusted) mean scores at the time point of interest by its pooled standard deviation. An effect size of 0.2 was considered to indicate a small effect, 0.5 a moderate effect and 0.8 a large effect.^29^ Where distribution of model residuals significantly differed from normality, models were administered (and effect sizes calculated) using transformed data.

Statistical analyses were undertaken using SPSS version 29 for Windows, supplemented where required by Stata SE version 16.0 for Windows.

## Results

**Fig. 1:**
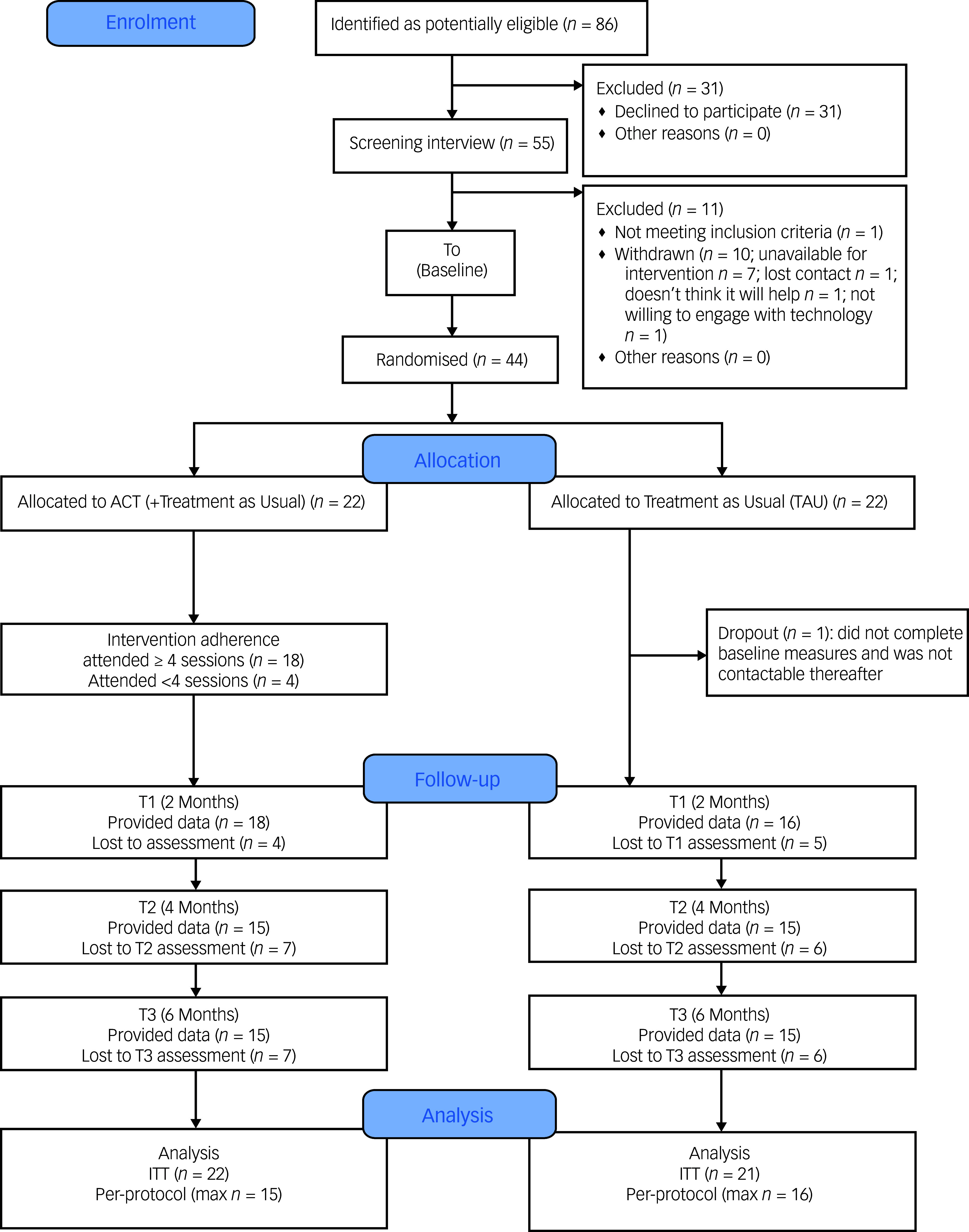
CONSORT (Consolidated Standards of Reporting Trials) diagram for participants. ITT, intention to treat; max *n*, maximum number of participants at any follow-up (for the acceptance and commitment therapy (ACT) (plus TAU) condition, this included only those who had attended ≥4 intervention sessions).

Participants were recruited from 7 November 2022 to 30 October 2023, during which time 86 patients were referred to the trial. Of these, 31 (36.0%) immediately declined involvement in the trial. Considering referrals by clinic type, 18 were from memory clinics (20.9%; ten declined to participate (55.6%)), 26 from cognitive neurology (30.2%; nine declined to participate (34.6%)) and 42 from neuropsychiatry (48.8%; 12 declined to participate (28.6%)). Therefore, 55 (64.0%) of all those referred attended an online screening interview. One of the 55 did not meet inclusion criteria, and ten (18.2%) withdrew, most commonly as they could not commit to attendance on the scheduled intervention dates (*n* = 7, 70.0%). Forty-four participants were randomised (participant flow, Fig. 1), corresponding to a participation rate of 51.1% (95% CI 40.8%, 61.5%).

Of the 22 patients randomised to ACT + TAU, *n* = 18 (81.8%; 95% CI 61.5%, 92.7%) attended at least four of the five sessions (*n* = 8 attended all five sessions). Four participants randomised to ACT + TAU did not meet the standard for successful adherence (18.2%).

Outcome data were available for 34 of 44 (77.3%) participants at 2 months, 30 of 44 (68.2%) at 4 months and 30 of 44 (68.2%) at 6 months. More than half of the participants (24 of 44, 54.5%) completed follow-up measures at all time points, and data from at least one follow-up were available for 38 of 44 (86.4%) participants. At each follow-up, measure completion rate was comparable between conditions. There was no association between participants’ pre-treatment scores on measure and attrition at follow-up, except for contentment with memory at 4-month follow-up (completed measures: mean 16.57, s.d. = 9.46; did not complete measures: mean 25.15, s.d. = 9.23; Hedges’ *g* = 0.90) and perceived memory ability at 6-month follow-up (completed measures: mean 26.35, s.d. = 14.99; did not complete measures: mean 34.74, s.d. = 16.98; Hedges’ *g* = 0.51); this suggested that participants with greater baseline memory contentment or ability were less likely to complete follow-up measures. The completion rates for the AD-SUS and EQ-5D-5L at baseline were between 95% and 100%, falling to between 63–68% at follow-up. Rates were slightly higher for the shorter EQ-5D-5L measure compared with the longer AD-SUS.

### Participant demographic and baseline clinical characteristics

Sociodemographic data, clinical information and screening measure scores of participants are shown in Table 1. One-quarter of participants were receiving antidepressant medication, whereas approximately one in six were taking an antiepileptic drug (mostly pregabalin for co-morbid pain and/or anxiety); such medications tended to be more common in participants in the TAU condition. Comorbid functional neurological disorder was reported by two participants in the TAU group.

**Table 1:** Sociodemographic information, clinical data and screening scores for anxiety and depression

Variable	ACT + TAU (*n* = 22)	TAU (*n* = 22)	Total
Age in years, mean (s.d.)	48.75 (13.84)	51.45 (12.00)	50.10 (12.87)
Gender, *n* (%)			
Female	14 (63.6)	13 (59.1)	27 (61.4)
Male	8 (36.4)	9 (40.9)	17 (38.6)
Ethnicity, *n* (%)			
Asian/Asian British	5 (22.7)	1 (4.5)	6 (13.6)
Arab/Arab British	0 (0.0)	1 (4.5)	1 (2.3)
Black/Black British	1 (4.5)	0 (0.0)	1 (2.3)
Dual heritage	0 (0.0)	2 (9.1)	2 (4.5)
White	16 (72.7)	18 (81.8)	34 (77.3)
White British	12 (54.5)	13 (59.1)	25 (56.8)
White Irish	1 (4.5)	1 (4.5)	2 (4.5)
White other	3 (13.6)	4 (18.2)	7 (15.9)
Employed, *n* (%)	13 (59.1)	13 (59.1)	26 (59.1)
Full-time employment	9 (40.9)	8 (36.4)	17 (38.6)
Part-time employment	4 (18.2)	5 (22.7)	9 (20.5)
Comorbid conditions, *n* (%)	15 (68.2)	17 (77.3)	32 (72.7)
Anxiety disorder	3 (13.6)	4 (18.2)	7 (15.9)
Arthritis	1 (4.5)	5 (22.7)	6 (13.6)
Cardiovascular disease	2 (9.1)	3 (13.6)	5 (11.4)
Chronic pain	1 (4.5)	5 (22.7)	6 (13.6)
Depressive disorder	0 (0.0)	2 (9.1)	2 (4.5)
Diabetes	3 (13.6)	1 (4.5)	4 (9.1)
Epilepsy	0 (0.0)	2 (9.1)	2 (4.5)
FND	0 (0.0)	2 (9.1)	2 (4.5)
Migraine	3 (13.6)	3 (13.6)	6 (13.6)
PTSD	2 (9.1)	1 (4.5)	3 (6.8)
Spinal disorder symptoms^a^	1 (4.5)	3 (13.6)	4 (9.1)
Other^b^	13 (59.1)	12 (54.5)	25 (56.8)
Current medications			
Antiseizure	2 (9.1)	5 (22.7)	7 (15.9)
Antidepressant	2 (9.1)	9 (40.9)	11 (25.0)
Antidiabetic	3 (13.6)	2 (9.1)	5 (11.4)
Antihistamine	2 (9.1)	2 (9.1)	4 (9.1)
Antipsychotic	1 (4.5)	0 (0.0)	1 (2.3)
Asthma	1 (4.5)	3 (13.6)	4 (9.1)
Cardiovascular	6 (27.3)	7 (31.8)	13 (29.5)
Hormone treatment	2 (9.1)	6 (27.3)	8 (18.2)
NSAID	2 (9.1)	1 (4.5)	3 (6.8)
Opioid	1 (4.5)	4 (18.2)	5 (11.4)
Sedative	1 (4.5)	1 (4.5)	2 (4.5)
Other^c^	8 (36.4)	7 (31.8)	15 (34.1)
Signposted to IAPT^d^	2 (9.1)	4 (18.2)	6 (13.6)
Depression and/or anxiety, mean (s.d.)			
PHQ-9 (0–27)	8.14 (4.16)	10.41 (5.38)	9.27 (4.87)
GAD-7 (0–21)	7.23 (4.50)	8.50 (6.62)	7.91 (5.71)
PHQ-9 moderate/severe (score ≥ 10), *n* (%)	7 (31.8)	14 (63.6)	21 (47.7)
GAD-7 probable anxiety disorder (score ≥ 8), *n* (%)	10 (45.5)	12 (54.5)	22 (50.0)

ACT, acceptance and commitment therapy; TAU, treatment as usual; PTSD, post-traumatic stress disorder; FND, functional neurological disorder; NSAID, non-steroidal anti-inflammatory drug; IAPT, Improving Access to Psychological Therapies; PHQ-9, nine-item Patient Health Questionnaire-9; GAD-7, Generalized Anxiety Disorder 7.

a. Spinal disorder symptoms did not include osteoarthritis.

b. Other comorbid conditions included anaemia, asthma, irritable bowel syndrome, long COVID-19, Ehlers–Danlos syndrome, tinnitus, dyslexia, glaucoma, psoriasis and colitis.

c. Other medication included proton-pump inhibitors, stimulants, selective serotonin receptor agonists (for migraine treatment), steroid treatment and anti-nausea medication.

d. Of those signposted to IAPT, two TAU participants were being treated, one TAU participant was waiting to be seen, two participants (one ACT + TAU participant and one TAU) had been discharged and the status of one ACT + TAU participant was not reported.

### Outcome measures

Table 2 presents the estimated means and treatment effect sizes for the primary and secondary outcome measures. For the AAQ-II, WHODAS 2.0, MMQ strategy, PHQ-9 and GAD-7, decreasing scores over time represent improvements; whereas for the EuroQol visual analogue scale (EQ-VAS), EQ-5D-5L, MMQ contentment and MMQ ability, increasing scores reflect improvements on the ICECAP. For all outcome measures, positive effect sizes represent an improvement in symptoms in ACT + TAU participants relative to TAU participants.

**Table 2:** Adjusted descriptive statistics and between-group effect size estimates for primary and secondary outcome measures (intention-to-treat approach)

Outcome measures	TAU Mean (s.e.)	ACT + TAU versus TAU Mean (s.e.)	Effect size^a^ (95% CI)
Primary outcome			
AAQ-II score (7–49)			
Baseline	21.50 (2.04)	20.86 (2.08)	
Post-treatment	21.72 (2.52)	22.12 (2.53)	0.10 (−0.48, 0.69)
Follow-up at 16 weeks	19.89 (2.07)	21.90 (2.15)	0.26 (−0.33, 0.85)
Follow-up at 26 weeks	18.71 (2.24)	22.06 (2.29)	0.39 (−0.20, 0.98)
Secondary outcomes			
Health-related quality of life and functioning			
WHODAS 2.0 total score (0–100)^b^			
Baseline	34.31 (4.68)	39.67 (4.79)	
Post-treatment	32.79 (4.73)	37.23 (4.86)	−0.05 (−0.64, 0.54)
Follow-up at 16 weeks	32.65 (4.81)	37.45 (4.90)	0.07 (−0.52, 0.66)
Follow-up at 26 weeks	31.27 (4.81)	41.92 (4.92)	0.30 (−0.29, 0.89)
ICECAP (0–20)			
Baseline	13.46 (0.63)	13.19 (0.65)	
Post-treatment	13.31 (0.65)	13.29 (0.68)	−0.08 (−0.67, 0.50)
Follow-up at 16 weeks	13.30 (0.68)	13.09 (0.69)	−0.02 (−0.61, 0.57)
Follow-up at 26 weeks	14.21 (0.68)	12.90 (0.69)	0.37 (−0.22, 0.96)
EQ-5D-5L total score (−0.285 to 1.000)			
Baseline	0.725 (0.063)	0.703 (0.064)	
Post-treatment	0.743 (0.064)	0.751 (0.066)	0.05 (−0.54, 0.63)
Follow-up at 16 weeks	0.749 (0.066)	0.685 (0.067)	0.22 (−0.37, 0.81)
Follow-up at 26 weeks	0.759 (0.066)	0.716 (0.068)	0.24 (−0.35, 0.83)
EQ-VAS score (0–100)			
Baseline	59.32 (4.79)	56.52 (4.89)	
Post-treatment	63.43 (4.97)	56.75 (5.16)	0.17 (−0.42, 0.76)
Follow-up at 16 weeks	67.35 (5.19)	52.66 (5.21)	0.52 (−0.08, 1.12)
Follow-up at 26 weeks	67.15 (5.11)	54.95 (5.20)	0.41 (−0.18, 1.01)
Subjective cognitive symptoms			
MMQ contentment score (0–72)			
Baseline	21.36 (2.12)	16.86 (2.17)	
Post-treatment	24.73 (2.38)	18.87 (2.49)	0.13 (−0.45, 0.72)
Follow-up at 16 weeks	28.67 (2.36)	17.00 (2.40)	0.71 (0.10, 1.31)
Follow-up at 26 weeks	28.28 (2.28)	18.22 (2.34)	0.55 (−0.05, 1.15)
MMQ ability score (0–80)			
Baseline	33.27 (3.51)	28.29 (3.60)	
Post-treatment	33.16 (3.52)	27.80 (3.63)	0.02 (−0.56, 0.61)
Follow-up at 16 weeks	36.02 (3.73)	28.10 (3.81)	0.18 (−0.41, 0.76)
Follow-up at 26 weeks	33.84 (3.58)	28.62 (3.67)	0.01 (−0.57, 0.60)
MMQ strategy score 0–76			
Baseline	37.86 (2.76)	39.57 (2.82)	
Post-treatment	40.13 (3.02)	40.10 (3.15)	−0.13 (−0.72, 0.46)
Follow-up at 16 weeks	39.69 (2.87)	40.67 (2.89)	−0.06 (−0.64, 0.53)
Follow-up at 26 weeks	38.88 (2.43)	42.52 (2.48)	0.15 (−0.44, 0.73)
Depression and/or anxiety			
PHQ-9 score (0–27)			
Baseline	8.55 (1.17)	9.29 (1.19)	
Post-treatment	8.18 (1.21)	10.13 (1.25)	0.23 (−0.36, 0.82)
Follow-up at 16 weeks	7.98 (1.24)	10.79 (1.26)	0.40 (−0.20, 0.99)
Follow-up at 26 weeks	7.54 (1.24)	10.74 (1.26)	0.47 (−0.12, 1.07)
GAD-7 score (0–21)			
Baseline	7.59 (1.17)	5.38 (1.20)	
Post-treatment	5.31 (1.20)	6.50 (1.25)	0.63 (0.03, 1.23)
Follow-up at 16 weeks	6.03 (1.16)	6.76 (1.19)	0.55 (−0.05, 1.15)
Follow-up at 26 weeks	6.35 (1.19)	7.58 (1.22)	0.64 (0.04, 1.24)

ACT, acceptance and commitment therapy; TAU, treatment as usual; AAQ-II, Acceptance and Action Questionnaire II; WHODAS 2.0, World Health Organization Disability Assessment Schedule version 2.0; ICECAP, Investigating Choice Experiments Capability Measure for Adults; EQ-5D-5L, five-dimension five-level EuroQol health scale; EQ-VAS, EuroQol visual analogue scale (current overall health rating (today)); MMQ, Multifactorial Memory Questionnaire; PHQ-9, nine-item Patient Health Questionnaire; GAD-7, Generalized Anxiety Disorder 7.

a. Effect sizes were calculated using Hedges’ *g*; positive effect sizes represent an improvement in symptoms (for MMQ strategy score, reduced strategy use was considered to indicate improvement, given the negative correlation between strategy use and memory satisfaction and/or ability). Effect sizes for WHODAS 2.0, EQ-5D-5L total score and MMQ strategy score were calculated using transformed data; raw (adjusted) mean data are shown.

b. WHODAS 2.0 mean (s.d.) values were calculated using complex scoring, based on item response theory-based scoring (which accounts for multiple levels of difficulty for each WHODAS item; Üstün, 2010^30^).

For the main outcome measure, psychological flexibility or inflexibility, participants in the intervention condition showed no change from baseline at 2-month follow-up, with mean decreases in psychological inflexibility of 1.6 and 2.8 points at the 4- and 6-month follow-ups, respectively. Between-group effect sizes for (change in) psychological flexibility (AAQ-II) were small at the 2-month (0.10) and 4-month (0.26) follow-ups and approached moderate at the 6-month follow-up (0.39).

Across measures of health-related quality of life and functional disability, there was little evidence for potential treatment benefit of ACT + TAU at the 2- and 4-month follow-ups. The ACT + TAU group reported improved overall health on the EQ-VAS at follow-ups (mean increases of between 4 and 8 points), with between-group effect sizes at 4- and 6-month follow-ups that were moderate in magnitude (0.41–0.52). Participants receiving ACT + TAU reported increased contentment with their memory at 4 and 6 months, with moderate between-group effect sizes at each follow-up (0.71 and 0.55, respectively). However, participants in both conditions reported little change in perceived memory ability or use of memory strategies at follow-ups. The most consistent signal of efficacy for ACT + TAU was for measures related to mood, most notably anxiety, for which between-group effect sizes ranged from 0.55 to 0.64 at follow-ups.

In terms of subjective improvement (Table 3), at 6-month follow-up, two-thirds (10 of 15) of the ACT + TAU group reported their condition had improved, whereas only one participant in the TAU condition did so.

**Table 3:** Clinical Global Impression Scale scores at 6-month follow-up

	ACT + TAU (*n* = 15)	TAU (*n* = 15)
Degree of improvement	Mean (s.d.)	Mean (s.d.)
Perceived change in condition from beginning to now (1–7)	3.33 (1.11)	4.73 (0.96)
	*n* (%)	*n* (%)
Very much improved	0 (0.0)	0 (0.0)
Much improved	3 (20.0)	0 (0.0)
Minimally improved	7 (46.7)	1 (6.7)
No change	3 (20.0)	6 (40.0)
Minimally worse	1 (6.7)	4 (26.7)
Much worse	1 (6.7)	4 (26.7)
Very much worse	0 (0.0)	0 (0.0)
Good outcome^a^	10 (66.7)	1 (6.7)

ACT, acceptance and commitment therapy; TAU, treatment as usual.

a. A rating of ‘improved’ or ‘much improved’ for perceived change in condition was considered to indicate a ‘good outcome’.

Table 4 shows mean (s.e.) values for outcome measures at each follow-up (adjusted for pre-treatment scores) for per-protocol participants. Differences between participants in the ACT + TAU and TAU conditions in terms of AAQ-II scores were moderate in size at 4- and 6-month follow-ups, whereas between-group effect sizes indicated small-to-moderate gains in the ACT + TAU group with respect to overall health (EQ-VAS), contentment with memory and mood (depression and anxiety) at 2-month follow-up, with moderate-to-large gains on these measures at subsequent follow-ups.

**Table 4:** Adjusted scores and between-group effect sizes for per-protocol participants completing measures at 2-, 4- and 6-month follow-ups

	Baseline		Follow-up (2 months)			Follow-up (4 months)			Follow-up (6 months)		
	ACT (*n* = 18)	TAU (*n* = 21)	ACT (*n* = 15)	TAU (*n* = 16)	ACT versus TAU	ACT (*n* = 12)	TAU (*n* = 15)	ACT versus TAU	ACT (*n* = 12)	TAU (*n* = 15)	ACT versus TAU
Outcome measures	Mean (s.e.)	Mean (s.e.)	Mean (s.e.)	Mean (s.e.)	Effect size (95% CI)	Mean (s.e.)	Mean (s.e.)	Effect size (95% CI)	Mean (s.e.)	Mean (s.e.)	Effect size (95% CI)
AAQ-II score (7–49)	20.17 (1.99)	20.86 (2.29)	20.50 (1.80)	22.41 (1.74)	0.20 (−0.40, 0.78)	15.88 (2.93)	21.42 (2.50)	0.57 (−0.03, 1.17)	17.74 (2.07)	22.34 (1.85)	0.39 (−0.20, 0.98)
WHODAS 2.0 score (0–100)	31.90 (5.18)	39.66 (3.88)	35.05 (4.37)	35.01 (3.57)	0.03 (−0.56, 0.61)	34.98 (5.79)	35.82 (3.58)	0.18 (−0.41, 0.77)	35.19 (5.97)	38.72 (3.72)	0.28 (−0.31, 0.87)
ICECAP score (5–20)	13.61 (0.64)	13.19 (0.60)	13.47 (0.42)	13.31 (0.41)	0.05 (−0.54, 0.63)	13.17 (0.73)	13.21 (0.63)	−0.01 (−0.60, 0.58)	14.38 (0.48)	12.89 (0.43)	0.48 (−0.11, 1.08)
EQ-VAS score (0–100)	59.17 (5.70)	56.52 (4.96)	62.31 (3.77)	55.71 (3.65)	0.28 (−0.31, 0.87)	66.23 (6.33)	52.37 (5.61)	0.52 (−0.08, 1.11)	65.03 (5.88)	55.25 (5.13)	0.38 (−0.22, 0.97)
EQ-5D-5L score (−0.285 to 1.000)	0.763 (0.068)	0.703 (0.066)	0.721 (0.064)	0.746 (0.048)	0.07 (−0.52, 0.66)	0.733 (0.068)	0.663 (0.074)	0.28 (−0.31, 0.87)	0.717 (0.078)	0.746 (0.059)	0.10 (−0.49, 0.69)
MMQ contentment score (0–72)	22.61 (2.20)	16.86 (2.25)	24.67 (2.88)	20.40 (2.47)	0.39 (−0.20, 0.98)	28.00 (3.57)	16.20 (1.84)	1.20 (0.56, 1.84)	26.25 (2.99)	18.13 (1.92)	0.85 (0.23,1.46)
MMQ ability score (0–80)	36.11 (3.86)	28.29 (3.28)	33.54 (3.55)	31.18 (2.72)	0.15 (−0.44, 0.74)	36.71 (4.41)	28.81 (3.09)	0.46 (−0.13, 1.06)	33.35 (3.96)	31.39 (3.05)	0.11 (−0.48, 0.70)
MMQ strategy score (0–76)	34.28 (2.19)	39.57 (2.87)	37.70 (2.38)	37.79 (2.31)	−0.004 (−0.59, 0.58)	40.11 (2.90)	39.32 (2.48)	−0.08 (−0.66, 0.51)	37.54 (32.06)	41.37 (1.84)	0.34 (−0.26, 0.93)
PHQ-9 score (0–27)	8.00 (1.06)	9.29 (1.15)	8.25 (1.00)	10.14 (0.97)	0.35 (−0.25, 0.94)	7.78 (1.27)	11.05 (1.05)	0.60 (−0.002, 1.20)	7.36 (1.39)	10.45 (1.33)	0.56 (−0.03, 1.16)
GAD-7 score (0–21)	7.00 (1.16)	5.38 (1.12)	4.16 (0.93)	7.17 (1.23)	0.57 (−0.03, 1.17)	3.51 (0.89)	7.33 (1.31)	0.68 (0.08, 1.29)	4.13 (0.92)	8.10 (1.23)	0.76 (0.15, 1.37)

ACT, acceptance and commitment therapy; TAU, treatment as usual; AAQ-II, Acceptance and Action Questionnaire II; WHODAS 2.0, World Health Organization Disability Assessment Schedule version 2.0; ICECAP, Investigating Choice Experiments Capability Measure for Adults; EQ-5D-5L, five-dimension five-level EuroQol health scale; EQ-VAS, EuroQol visual analogue scale (current overall health rating (today)); MMQ, Multifactorial Memory Questionnaire; PHQ-9, nine-item Patient Health Questionnaire; GAD-7, Generalized Anxiety Disorder 7.

a. Effect sizes were calculated using Hedges’ *g*. Positive effect sizes represent an improvement in symptoms (for MMQ strategy scores, reduced strategy use was considered to indicate improvement, given the negative correlation between strategy use and memory satisfaction and/or ability). Effect sizes for WHODAS 2.0, EQ-5D-5L total score and MMQ strategy score were calculated using transformed data; raw (adjusted) mean data are shown.

### Intervention acceptability

On the post-treatment feedback form, almost two-thirds (9 of 15) of participants in the intervention group reported at 6-month follow-up that they were either completely satisfied or satisfied with their treatment (Table 5). One participant in the ACT + TAU condition reported that they were unsatisfied. No participant in the TAU condition indicated that they were satisfied, and just over half (8 of 15) reported that they were unsatisfied with their care.

**Table 5:** Satisfaction with functional cognitive disorder (FCD) care

Degree of satisfaction	ACT + TAU (*n* = 14)	TAU (*n* = 15)
Satisfaction with overall care received for FCD (1–5), mean (s.d.)	3.79 (0.89)	2.40 (0.63)
Intervention acceptability, *n* (%)		
Very unsatisfied	0 (0.0)	1 (6.7)
Unsatisfied	1 (7.1)	7 (46.7)
Neutral	4 (28.6)	7 (46.7)
Satisfied	6 (42.9)	0 (0.0)
Very satisfied	3 (21.4)	0 (0.0)
Satisfied/very satisfied (combined)	9 (64.3)	0 (0.0)

ACT, acceptability and commitment therapy; TAU, treatment as usual.

### Adverse events

There were six adverse events involving three participants, and no serious adverse events over the course of the study. Of the six adverse events reported, five (83.3%) were experienced by participants in the TAU condition. One (16.7%) adverse event of moderate severity was reported by a participant in the ACT + TAU condition. None was attributable to participation in the study.

### Feasibility criteria

Of the predefined feasibility criteria, the study partially met the recruitment rate criterion (51.1% against a target of 70% or above) and fully met the others: intervention adherence (81.8% against a target of 75% or above), intervention acceptability (60% reported being satisfied or very satisfied, against a target of 50% or above) and signal of efficacy (0.39 change in AAQ-II score (and other measures) demonstrating a trend). See Table 6 for details.

**Table 6:** Feasibility criteria standards, actual outcomes and whether standards were met, partially met or unmet

Criterion	Proposed threshold	Outcome	Feasibility
Recruitment rate	If 70% or above agree to recruitment, continue to main study without modifications If 50–70% agree, consider ways to increase the recruitment rate (future definitive trial is feasible with modifications) If less than 50% agree, feasibility has not been demonstrated (future definitive trial is not feasible)	44 participants were randomised of 86 referred to the study (51.1%; 95% CI 40.8%, 61.5%)	Partially met
Intervention adherence	Feasibility will be considered to have been demonstrated if more than 75% complete four or more ACT sessions (continue to main study without modifications) If adherence is 50–75%, consider ways to improve engagement (future definitive trial is feasible with modifications) If adherence is less than 50%, feasibility has not been demonstrated (future definitive trial is not feasible)	18 of 22 participants attended four or more sessions 81.8% (95% CI 61.5%, 92.7%)	Met
Intervention acceptable	If the majority of participants report being satisfied or very satisfied with the intervention (score of 4 or 5) on five-point Likert scale (continue to main study without modifications) If majority score is 3, consider ways to improve the acceptability of the intervention (future definitive trial is feasible with modifications) If majority score is less than below 3, feasibility has not been demonstrated (future definitive trial is not feasible)	Nine of 15 participants (60%) reported at 6-month follow-up that they were either satisfied (*n* = 6) or very satisfied (*n* = 3)	Met
Signal of efficacy	AAQ-II score	Effect size at 26 weeks follow-up: 0.39 (−0.20, 0.98)	Met

ACT, acceptability and commitment therapy; AAQ-II, Acceptance and Action Questionnaire II.

## Discussion

This randomised controlled feasibility trial partially met the criteria set for recruitment and fully met those for adherence, acceptability and demonstration of signal of efficacy. Given the relatively high numbers of patients with FCD seen in cognitive neurology, neuropsychiatry and memory clinics,^2^ and the successful recruitment of more than 50% of those referred, running a larger multicentre definitive RCT seems feasible. However, either further work will be required to increase the recruitment rates, or we must accept that the proposed recruitment rate was over-optimistic and modify the recruitment period accordingly. The results of this feasibility study compare favourably with those of similar trials in functional neurological disorders. For example, in one study, 34.4% of those referred for cognitive–behavioural therapy for functional seizures were ultimately recruited,^31^ and in another, 28.6% of those with functional movement disorders were recruited from specialist neurophysiotherapy services.^32^ However, a recent feasibility study of cognitive–behavioural therapy versus neurorehabilitation for FCD following concussion^33^ reported 86% recruitment, although participants in that study were either members of a cohort previously involved in concussion research who had signed an additional form requesting to be contacted about future studies or were recruited from specialist concussion clinics; therefore, they may have represented a highly motivated group not readily comparable with the individuals approached from our recruiting sites, which included generic memory clinics. It may also be necessary to increase awareness of FCD among memory clinic staff, as they are likely to mischaracterise patients with FCD as having mild cognitive impairment,^1^ a precursor of dementia. This misdiagnosis, however, is liable to exacerbate FCD symptoms and associated distress, as well as impeding access to potentially beneficial interventions. Engaging leaders in dementia research and clinical services and producing easy-to-access training materials could improve knowledge, and a large multicentre clinical trial will itself also enhance familiarity with the diagnosis.

The recruitment from a range of clinic settings indicates that the sample recruited was representative of those with FCD, supporting the generalisability of the findings. Furthermore, of those interested in participating, 80% were randomised into the study, and they remained engaged, with good rates of completion of outcome measures and the ACT intervention itself. A larger trial could allow greater flexibility when offering intervention dates, thereby improving the enrolment rate, as nearly one-fifth of those offered the participation could not commit to the times and/or dates on offer.

The satisfaction with care of those in the ACT + TAU group was in contrast to that of those receiving only the current standard treatment. Likewise, the active intervention group generally reported being ‘minimally improved’ or ‘much improved’, whereas just one participant in the TAU group perceived a minimal improvement. CGI scores of ACT + TAU participants improved only modestly; however, this is perhaps not surprising, as ACT purposefully contrasts the suffering attributable to the condition itself to responses to it and aims to address only the latter. This perhaps explains the apparent dissociation between the satisfaction and subjective improvement scores. In keeping with this, the satisfaction of the ACT + TAU participants with their memory did improve, whereas their subjective memory function scores did not. By contrast, those in the TAU condition remained highly dissatisfied with their memory function. The intervention proved safe, with no adverse events or serious adverse events attributable to either ACT or TAU.

Completion rates for the outcome measures dropped over the course of the 6-month follow-up period, despite financial inducement at each time point. This feasibility study was also designed to identify a signal of efficacy within the variety of outcome measures used. The AAQ-II was pragmatically chosen as the primary outcome measure and did demonstrate a modest difference between the two groups at 4 and 6 months, especially when assessing only those who attended four or more sessions as per the intervention protocol. It is not clear whether such relatively modest changes between baseline and 6-month follow-up scores represent a clinically significant decrease in psychological inflexibility; however, there was a trend of improvement from baseline in the intervention group, in contrast to a deterioration in the TAU group. This was in conjunction with the moderate gains on measures of overall health (EQ-VAS), memory satisfaction and mood, which were apparent immediately after the ACT intervention and maintained at 4 and 6 months. This improvement in scores across a broad range of measures is supportive of the modest improvement in psychological flexibility being associated with clinically meaningful change, if not necessarily being the driver of the changes. It remains an open question whether the AAQ-II should be the primary measure in a future definitive RCT, however. Given the elevated rates of mood disorders in the cohort, a future trial should analyse the effects of depression and anxiety severity on outcomes.

There were some limitations to this study and its findings. Being a feasibility trial, it was not designed or powered to identify any treatment effect of the ACT intervention. Also, any differences between groups could reflect performance bias (as the groups were treated differently and unblinded to their intervention arm), differences between the groups at baseline (as the control group showed elevated levels of depression), randomness or some combination of these factors, among others. The higher rate of antidepressant use in the TAU group reinforces the point that this may have been a more unwell group, and this could be reflected in the 6-month outcomes. However, the imbalance between the two trial conditions in terms of PHQ-9 scores at the screening stage had disappeared by the time baseline measures were completed. A larger trial would be better able to ensure equality at baseline between the two arms. Furthermore, the participants were all unblinded to treatment, and those randomised to the TAU arm may have experienced greater frustration and hopelessness caused by being denied an active intervention for their symptoms, creating expectancies about outcome in both groups. The completion rates for outcome measures in both groups dropped below 70% after 2 months, despite frequent reminders; this limited the interpretation of findings and might indicate that the measures were overly onerous to complete. A future trial should revisit the question of which measures are necessary, with input from a PPI group.

As TAU was delivered by the referring teams, it is likely that there was significant variation in what exactly this included. Qualitative research conducted as part of the study (in preparation) suggests that not all participants were informed clearly of their diagnosis, although memory-related factors may have limited recall. Nearly one-third of potential participants declined further involvement upon first contact with the research team; however, we know nothing of their characteristics. It is unclear how to make a future trial more appealing to this group, but improved explanation of the diagnosis when it is made^17^ and description of the trial are likely to be beneficial. A strength of the study was the low rate of participants excluded following screening, so the sample is likely to reflect that found in clinic settings rather than being highly selective.

In summary, we report on a novel brief group intervention which can be delivered remotely and is regarded as acceptable and potentially beneficial. It is not clear which outcome measure should be used as a primary outcome, however. The results support the need for a larger definitive multicentre RCT of this intervention.

## Data Availability

The data that support the findings of this study are available on request from the corresponding author, N.P.
